# Resuscitative endovascular balloon occlusion of the aorta may increase the bleeding of minor thoracic injury in severe multiple trauma patients: a case report

**DOI:** 10.1186/s13256-017-1511-0

**Published:** 2017-12-14

**Authors:** Takaaki Maruhashi, Hiroaki Minehara, Ichiro Takeuchi, Yuichi Kataoka, Yasushi Asari

**Affiliations:** 1 Department of Emergency and Critical Care Medicine, Kitasato University School of Medicine, 1-15-1 Kitasato, Minami-ku, Sagamihara, Kanagawa 252-0375 Japan; 2 Department of Orthopedic Surgery, Kitasato University School of Medicine, 1-15-1 Kitasato, Minami-ku, Sagamihara, Kanagawa 252-0375 Japan

**Keywords:** Resuscitative endovascular balloon occlusion of the aorta, Coagulopathy, Multiple trauma, Massive hemothorax, Pelvic fracture

## Abstract

**Background:**

The resuscitative endovascular balloon occlusion of the aorta, because of its efficacy and feasibility, has been widely used in treating patients with severe torso trauma. However, complications developing around the site proximal to the occlusion by resuscitative endovascular balloon occlusion of the aorta have almost never been studied.

**Case presentation:**

A 50-year-old Japanese woman fell from a height of approximately 10 m. At initial arrival, her respiratory rate was 24 breaths/minute, her blood oxygen saturation was 95% under 10 L/minute oxygenation, her pulse rate was 90 beats per minute, and her blood pressure was 180/120 mmHg. Mild lung contusion, hemopneumothorax, unstable pelvic fracture, and retroperitoneal bleeding with extravasation of contrast media were observed in initial computed tomography. As her vital signs had deteriorated during computed tomography, a 7-French aortic occlusion catheter (RESCUE BALLOON®, Tokai Medical Products, Aichi, Japan) was inserted and inflated for aortic occlusion at the first lumbar vertebra level and transcatheter arterial embolization was performed for the pelvic fracture. Her bilateral internal iliac arteries were embolized with a gelatin sponge; however, the embolized sites presented recanalization as coagulopathy appeared. Her bilateral internal iliac arteries were re-embolized by n-butyl-2-cyanoacrylate. The balloon was deflated 18 minutes later. After embolization, repeat computed tomography was performed and a massive hemothorax, which had not been captured on arrival, had appeared in her left pleural cavity. Thoracotomy hemostasis was performed and a hemothorax of approximately 2500 ml was aspirated to search for the source of bleeding. However, clear active bleeding was not captured; resuscitative endovascular balloon occlusion of the aorta may have been the cause of the increased bleeding of the thoracic injury at the proximal site of the aorta occlusion.

**Conclusions:**

It is necessary to note that the use of resuscitative endovascular balloon occlusion of the aorta may increase bleeding in sites proximal to occlusions, even in the case of minor injuries without active bleeding at the initial diagnosis.

## Background

Temporary aortic occlusion may be performed prior to definitive hemostasis for trunk injury accompanied by hemorrhagic shock in which bleeding control is difficult. Aortic occlusion was conventionally performed by cross-clamping the aorta after resuscitative thoracotomy. In recent years, resuscitative endovascular balloon occlusion of the aorta (REBOA) has been widely used to treat patients with severe torso trauma. It has been reported that invasion by REBOA is much lower than that in the case of cross-clamping, and although there are no significant differences in the time required for occlusion, the use of REBOA correlates with higher survival rates [1]. Therefore, cross-clamping after thoracotomy has been replaced with the aortic occlusion technique because of the latter’s greater efficacy and feasibility.

Although great benefits are expected as described above, complications related to catheter insertion are known as common complications accompanying REBOA: vascular injury, aorta dissection, aberration of the catheter, and organ ischemia of the site distal to the occlusion accompanied by prolonged occlusion. However, complications developing around the site proximal to the occlusion, such as the possibility of increase in bleeding in the site proximal to the occlusion and the side effects of hyperperfusion, have hardly been studied to date. A systematic review [2] of REBOA, which included 61 articles and 1355 patients, showed that complications of the site proximal to the occlusion occurred only in one patient (0.07%) [3].

We experienced a case of severe multiple trauma with impending cardiac arrest, in which REBOA was used to avoid cardiac arrest, where REBOA possibly caused major bleeding from a thoracic injury that had been diagnosed as mild at initial arrival.

Here we report this case to raise a concern that the use of REBOA may cause unclarified bleeding complications at sites proximal to an occlusion.

## Case presentation

A 50-year-old Japanese woman fell from a height of approximately 10 m and was brought to our hospital by an ambulance. She had no remarkable medical and family history, was a social drinker, and a non-smoker of tobacco. Her marital status was stable with her husband. Her consciousness levels were 14 points (E4, V4, M6) according to the Glasgow Coma Scale at initial arrival. She complained of respiratory discomfort and low back pain, and was in an unrestful state. Her respiratory rate was 24 breaths/minute, blood oxygen saturation (SpO_2_) was 95% under 10 L/minute oxygenation, subcutaneous emphysema was recognized in the left side of her chest, and her breathing sounded weak. A hemoperitoneum was not detected on focused assessment with ultrasonography for trauma. Her pulse rate was 90 beats per minute (bpm) and her blood pressure was 180/120 mmHg as measured by an automatic blood pressure monitor. However, her capillary refill time was 3 seconds and radial artery pulsation was feeble with cold sweat in her extremities; hence, she was recognized as being in a state of shock. Her face swelled, and bleeding from her nasal and the oral cavities continued. A subcutaneous hematoma was found in her lumbar and her left femoral region. Her left elbow was deformed and swollen. Because she was in a restless state, accurate neurological assessments were difficult, but coarse paralysis of limbs was not observed.

**Fig. 1:**
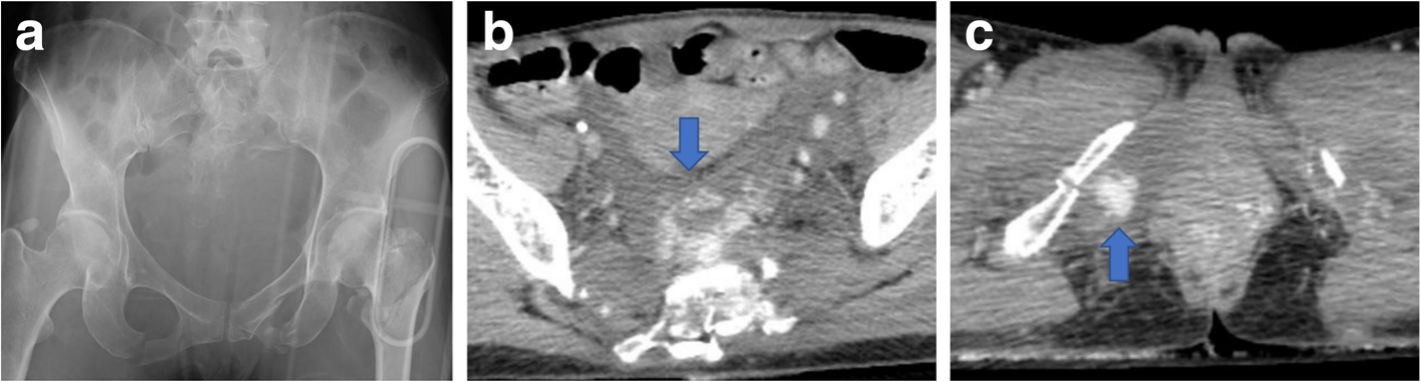
Pelvis X-ray (**a**) and contrast computed tomography (**b** and **c**) at the initial arrival. a Fractures of pubic bones, hipbones on both sides, and sacral bones are seen and there are unstable pelvic fractures. A massive retroperitoneal hematoma and extravasation of the contrast media on the anterior of sacrum (**b**; arrow) and right perineum (**c**; arrow) are recognized

First, a 28-French chest drain was inserted in her left thoracic cavity for the diagnosis of tension pneumothorax. Hemothorax was not recognized. A pelvis X-ray showed unstable pelvic fracture and a contrast-enhanced computed tomography (CT) pan scan was additionally performed. In the contrast CT, retroperitoneal bleeding with the extravasation of contrast media was recognized (Fig. 1). Other injuries and laboratory data on initial arrival are shown in Tables 1 and 2 respectively.

**Table 1:** Injury site and diagnosis of this case along with Abbreviated Injury Scale coding

Body region	Diagnosis
Head and neck	Brain contusion (brain stem, cerebrum)
Face	Mandible fracture
	Zygoma fracture
	Nose fracture
Chest	Multiple rib fractures
	Hemopneumothorax
	Thoracic spine fracture
Abdominal	Liver laceration
	Lumbar spine fracture
Extremities and pelvis	Femoral neck fracture
	Pelvic fracture
External	None

Injury severity score: 66

Probability of survival: 59.3%

**Table 2:** Laboratory data on initial arrival

Complete blood count		
WBC	4500	/μl
Neu	71.8	%
Lym	24.9	%
RBC	378	× 10^4^/μl
Hb	11.6	g/dl
Ht	34.4	%
Plt	22.5	× 10^4^/μl
Arterial blood gas (10 L/minute oxygenation)		
pH	7.451	
PO_2_	41.7	mmHg
PCO_2_	251.3	mmHg
HCO_3_^-^	28.4	mmol/l
BE	4.1	mmol/l
Lac	43.4	mg/dl
Chemistry		
TP	6.0	g/dl
Alb	3.8	g/dl
BUN	12.6	mg/dl
Cre	0.84	mg/dl
Na	135	mEq/l
K	3.3	mEq/l
Cl	97	mEq/l
Ca	9.1	mg/dl
AST	321	U/l
ALT	217	U/l
LDH	902	U/l
ALP	170	U/l
T-Bil	1.3	mg/dl
CPK	450	U/l
CRP	0.03	mg/dl
Coagulation		
APTT	30.3	seconds
PT	65	%
PT-INR	1.23	
Fib	171	mg/dl
FDP	80.5	μg/ml
D-dimer	40.16	μg/ml

*Alb* albumin, *ALP* alkaline phosphatase, *ALT* alanine aminotransferase, *APTT* activated partial thromboplastin time, *AST* aspartate aminotransferase, *BE* base excess, *BUN* blood urea nitrogen, *Ca* calcium, *Cl* chlorine, *CPK* creatine phosphokinase, *Cre* creatinine, *CRP* C-reactive protein, *FDP* fibrin degradation product, *Fib* fibrinogen, *Hb* hemoglobin, *HCO*_*3*_^*-*^ bicarbonate, *Ht* hematocrit, *K* potassium, *Lac* lactate, *LDH* lactate dehydrogenase, *Lym* lymphocyte, *Na* sodium, *Neu* neutrophil, *PCO*_*2*_ partial pressure of carbon dioxide, *Plt* platelets, *PO*_*2*_ partial pressure of oxygen, *PT* prothrombin time, *PT-INR* prothrombin time-international normalized ratio, *RBC* red blood cells, *T-Bil* total bilirubin, *TP* total protein, *WBC* white blood cells

**Fig. 2:**
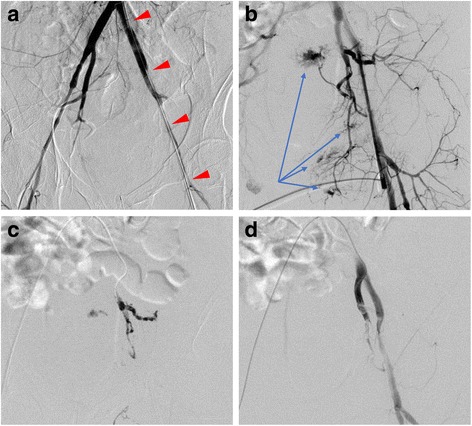
Transcatheter arterial embolization for the pelvic fracture. The contrast media extravasation ofthe internal iliac artery area is clear in computed tomography. First, the aortic occlusion catheter was inserted from the left femoral artery (arrowheads) and was embolized with a gelatin sponge from the origin portion of the bilateral internal iliac artery while the aorta was occluded at the first lumbar vertebra level. **a** Embolization of the lumbar artery and middle sacral artery was additionally performed and angiography from the sheath reinserted to the left femoral artery presented re-bleeding (arrow). **b** As it was difficult to arrest hemorrhage with a gelatin sponge, hemorrhage was arrested by embolizing additionally with n-butyl-2-cyanoacrylate (**c** and **d**)

**Fig. 3:**
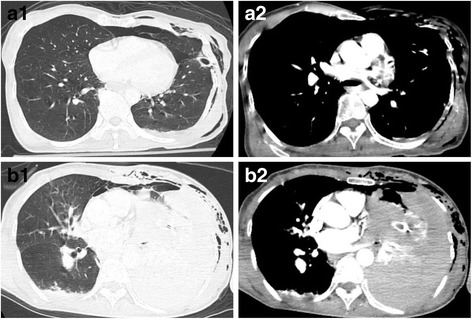
Comparison of the chest computed tomography images at initial arrival (**a1** and **a2**) and post-embolization (**b1** and **b2**). Pneumothorax and subcutaneous emphysema were recognized at the initial diagnosis by computed tomography and, therefore, a chest drain was inserted. Pulmonary contusion was extremely minor and active bleeding such as the intercostal artery injury was not captured (**a1** and **a2**). A large hemothorax appeared on reexamination by computed tomography after transcatheter arterial embolization (**b1** and **b2**). Similar active bleeding was not observed at the initial diagnosis computed tomography. A large hemothorax of 2500 ml was observed in thoracotomy hemostasis. However, active bleeding requiring hemostasis treatment was not recognized. The inserted chest drain was occluded by blood clots

The injury severity score in this case was 66 and the probability of survival was calculated as 59.3%. Multiple rib fractures were seen, even though active bleeding from intercostal artery injury and pulmonary laceration were not noted at the initial visit. She had deteriorated during CT (blood pressure 67/42 mmHg, pulse rate 75 bpm), and blood transfusion was therefore started and intubation was performed. Transcatheter arterial embolization was performed for hemorrhagic shock with the pelvic fracture as the main bleeding source. While starting angiography, she did not respond to the massive blood transfusion and her systolic blood pressure was maintained at around 60 mmHg. Therefore, first, a 7-French aortic occlusion catheter (RESCUE BALLOON®, Tokai Medical Products, Aichi, Japan) was inserted from her left femoral artery. Her hemodynamics had improved and her systolic blood pressure was 90 mmHg due to REBOA (30 mL saline inflation into the balloon) at the first lumbar vertebra level. Next, transcatheter arterial embolization was started using the sheath inserted in her right femoral artery. Her bilateral internal iliac arteries were embolized from the origin portion with a gelatin sponge. Furthermore, we embolized the extravasation of contrast media of her middle sacral artery and lumbar artery with n-butyl-2-cyanoacrylate. Her blood pressure had been monitored by continuous invasive arterial measurement from her upper right brachial artery during interventional radiology. The systolic blood pressure under REBOA was intended to be controlled under 100 mmHg (so called permissive hypotension); however, it was actually in the range of 90 to 160 mmHg. After 18 minutes of occlusion, the balloon was deflated and the aortic occlusion catheter was removed. However, she was still hemodynamically unstable. The internal iliac artery angiography was performed again, and the site that had just been embolized, as described above, presented recanalization. Since coagulopathy was recognized as a complication, we interpreted that it would be difficult to restrain the hemorrhage with the gelatin sponge. Therefore, it was re-embolized from the origin portion of her bilateral internal iliac arteries by n-butyl-2-cyanoacrylate (Fig. 2). After embolization, retroperitoneal gauze packing and pelvic external skeletal fixation were additionally performed. Further, a repeat CT pan scan was performed, and a massive hemothorax had appeared in her left pleural cavity, which had not been captured at the first arrival (Fig. 3). As the inserted chest drain at the first arrival was detained between lobes, the amount of bleeding in the pleural cavity had not been reflected precisely. Thoracotomy hemostasis was performed and a hemothorax of approximately 2500 ml was aspirated to search for the source of the bleeding. However, clear active bleeding was not captured and only extremely minute bleeding from the rib fracture site, chest wall, and pulmonary contusion area was detected. Therefore, special hemostasis treatment was not needed. After a massive hematoma was removed, a chest drain was placed again and her chest was closed. Continuous blood transfusion was not needed before or after the operation and her postoperative hemodynamics became stable. The timeline of the initial treatment in our emergency department is shown in Table 3.

**Table 3:** The timeline of initial treatment in our emergency department

Time course after arrival at the hospital	Survey and treatment
0 min	Hospital arrival
5 min	Fluid resuscitation start
10 min	Left chest drain insertion
12 min	Focused assessment with ultrasonography for trauma
19 min	Chest and pelvis X-ray
44 min	Whole body CT scan
57 min	Transfusion start
72 min	Intubation
83 min	Endovascular treatments start
	Resuscitative endovascular balloon occlusion of the aorta
100 min	The bilateral internal iliac arteries were embolized
101 min	The balloon of the aortic occlusion catheter was deflated
	Retroperitoneal gauze packing
	Pelvic external skeletal fixation
600 min	Repeat CT scan
631 min	Thoracotomy hemostasis for left massive hemothorax

*CT* computed tomography, *min* minutes

The retroperitoneal packing gauze was removed on day 3. On day 24, the chest drain was removed without increasing the hemothorax. Complications accompanied by internal iliac artery occlusion, such as gluteal muscle necrosis, did not appear. She regained the ability to walk, and was moved to another hospital on day 154 for rehabilitation. She was discharged from the rehabilitation hospital 6 months after the injury, and her activities of daily living were almost independent.

## Discussion

This case was in a state of threatened cardiac arrest from hemorrhagic shock due to pelvic fracture, but cardiac arrest was avoided by REBOA. However, bleeding of the thoracic injury proximal to the occlusion increased over time, and additional thoracotomy hemostasis was required. The CT at the initial arrival had presented only mild lung contusion. Arterial hemorrhage that resulted in massive hemothorax in a short time was not shown. It is known that delayed massive hemothorax develops in 7.4% of blunt thoracic injuries with rib fracture, even if it is a minor injury in the initial diagnosis [4]. It usually develops from 18 hours to 6 days (3 days on an average) after injury [5]. Moreover, its cause is bleeding from the intercostal artery in most cases, but diaphragm injuries can also be the cause in some cases. In this study, the findings obtained by CT and open thoracotomy after the removal of the aortic occlusion did not show active bleeding, and intercostal aneurysms and diaphragm injuries were not confirmed. In a head and pelvic injury case treated by REBOA, Uchino *et al*. reported that a complication developed in the site proximal to the occlusion by REBOA in which minor intracranial bleeding was seen in the head CT at the initial diagnosis, wherein the bleeding suddenly increased and a cerebral hernia developed and the patient died [3]. In this case, REBOA excessively raised the perfusion pressure in the chest area proximal to the occlusion, and coagulopathy occurred, induced by trauma shock and trauma itself. Therefore, we considered that bleeding accompanied by extremely minor thoracic injury had increased unexpectedly.

Several methods to avoid complications while performing insertions have been reported, such as the insertion method with ultrasonography [6, 7], insertion training with a cadaver [8], and the use of smaller introducer sheaths for REBOA [9]. In fact, it has also been reported that a good outcome was obtained by REBOA in the course of prehospital care for severe pelvic fracture as it can be inserted safely without fluoroscopy [10].

However, only a few studies have focused on blood flow evaluation at the site proximal to the occlusion in an aortic occlusion, or discussed perfusion pressure in detail. Therefore, a clear standard does not exist for the best occlusion rate, or for bleeding control at a site distal from the occlusion without increasing bleeding at a site proximal to the occlusion. Furthermore, it has been reported that in an analysis of data from the Japan Trauma Data Bank, the use of REBOA increased mortality compared with that of cases without REBOA [11]. REBOA is a revival treatment required in an emergency situation, where there is no other way but to employ REBOA, and it is used at a stage when, it is assumed, detailed information about the lesion and the severity has not yet been grasped. Furthermore, since the frequency of its use is expected to rise in various situations both in and out of hospital for trauma and endogenous diseases in the future, it is necessary to study the evaluation of blood flow at the site proximal to occlusions, the optimal methods of monitoring such blood flow, and the optimal occlusion rates.

## Conclusions

A method for the evaluation of blood flow has not been established for sites proximal to occlusions by REBOA. We report this case to call attention to the use of REBOA which may increase bleeding in sites proximal to occlusions unexpectedly in multiple trauma with head or thoracic injuries, even in the case of minor injuries without active bleeding at the initial diagnosis.

## Abbreviations

Bpm: Beats per minute
CT: Computed tomography
REBOA: Resuscitative endovascular balloon occlusion of the aorta
